# Employment after spinal cord injury in the United States: predictors and barriers

**DOI:** 10.3389/fresc.2026.1737005

**Published:** 2026-06-15

**Authors:** Georgios Theotokatos, Cristina Sadowsky, Shannon Inches, Melissa M. Morrow, Michael Furtado, Lucas R. Burns, Reuben Escorpizo

**Affiliations:** 1 Department of Physiotherapy, School of Health and Care Sciences, University of West Attica, Athens, Greece; 2 International Center for Spinal Cord Injury, Kennedy Krieger Institute Baltimore, MD, United States; 3 Department of Physical Medicine and Rehabilitation, Johns Hopkins University School of Medicine, Baltimore, MD, United States; 4 Department of Physical Therapy and Rehabilitation Sciences, Sealy Center on Aging, University of Texas Medical Branch, Galveston, TX, United States; 5 Department of Physical Therapy, University of North Texas Health, Fort Worth, TX, United States; 6 Department of Rehabilitation and Movement Science, The University of Vermont, Burlington, VT, United States

**Keywords:** disability benefits, employment outcomes, functioning, ICF, perceived barriers, quality of life, spinal cord injury, vocational rehabilitation

## Abstract

**Introduction:**

Spinal cord injury (SCI) is a health condition that severely affects functioning, quality of life, social participation, and employment outcomes.

**Methods:**

This cross-sectional observational study analyzed data from 167 participants with SCI who lived in the United States and participated in the most recent International Spinal Cord Injury Survey (InSCI); it aimed to examine sociodemographic, injury, and support-related predictors of employment and to investigate the perceived barriers to work. Descriptive statistics and binary logistic regression were applied.

**Results:**

Overall, 67.7% of the participants were employed; those aged 30–39, had more years of education, and living with a partner showed higher odds of employment. Significant lower odds of employment were observed in those who had a shorter time since injury and were recipients of disability benefits. The most common barriers for the unemployed individuals were insufficient transportation services (82.5%), fear of losing benefits (33.8%) and inaccessible workplaces (11.3%).

**Discussion:**

Our findings suggest that employment after SCI is determined from individual characteristics and systemic barriers. The results highlight the importance of education, vocational rehabilitation, accessibility facilitators, and disability benefit policy-related reforms that promote equitable workforce participation, inclusion, and functioning in SCI.

## 1 Introduction

In 2019, there were approximately 0.9 million new cases of spinal cord injury (SCI) globally, and an estimated 20.6 million individuals were living with SCI (1, 2). Ding et al. also noted that high-income North America accounted for one of the largest burdens, with over 100,000 new cases annually and the highest prevalence worldwide, reflecting both population aging and the long-term disability associated with SCI. People with SCI face motor, sensory, and autonomic impairments that lead to substantial functioning limitations, alongside secondary health conditions like bladder/bowel management limitations, pain, spasticity, urinary tract infections, sexual dysfunction, pressure sores or respiratory problems. The presence and severity of these complications may depend on the lesion characteristics, including level (paraplegia/tetraplegia) and type (complete/incomplete). Despite recent trends showing declines in age-standardized incidence and prevalence, the absolute number of cases continues to rise due to population aging and growth, while secondary health issues and socioeconomic impact, making SCI a persistent global health challenge (3). Environmental factors can act as facilitators or barriers to the participation of people with SCI (4). Employment after SCI offers numerous financial, psychological, social, and health-related benefits (5). However, employment rates among individuals with SCI remain consistently lower than those of the general population across the globe (6, 7). In the United States, studies suggest that employment in individuals with SCI related neurologic deficits consistently show low rates of return to work, often ranging between 20% and 40% depending on time since injury and sociodemographic factors (8–10). United States data show that perceived barriers and facilitators, such as resources, self-perception of health, motivation, and disincentives, relate to labor-force participation (10).

This disparity is influenced by a range of factors serving as barriers or facilitators, including physical limitations, workplace accessibility, vocational counseling services, and social attitudes and beliefs. Recent evidence by Karcz et al. (11) showed that the subjective perspective can influence the complex sustainable employment status after SCI (11). Understanding the complexity and the interaction of these barriers is critical to improving employment outcomes. In response to this need, international efforts such as the International Spinal Cord Injury (InSCI) survey have sought to gather comprehensive data on the self-reported perspectives of individuals with SCI, based on the WHO's International Classification of Functioning, Disability and Health (ICF) (12).

Beyond clinical and economic perspectives, the right to work is recognized as a fundamental human right under the United Nations Convention on the Rights of Persons with Disabilities (CRPD) (13). The CRPD emphasizes non-discrimination, equality, and access to employment, offering a global goal to alleviate environmental and policy-related barriers and facilitate work integration, including people with SCI. Considering the United States, the Americans with Disabilities Act (ADA) (1990) was established to prohibit discrimination and ensure equal opportunity. Nevertheless, the structure of US federal disability benefits: the Social Security Disability Insurance (SSDI) and Supplemental Security Income (SSI), often creates a conflicting dynamic known as the ‘benefit cliff’ (14). Unlike systems in some other InSCI participating countries where partial disability benefits allow for gradual workforce reintegration, US policy imposes strict income thresholds (Substantial Gainful Activity). Exceeding these limits can result in the sudden termination of cash benefits and, critically, the loss of essential health coverage (Medicare/Medicaid) (14, 15). Consequently, understanding the self-reported perspective in the US requires investigating not just physical accessibility, but how these systemic policy designs are perceived by individuals as tangible barriers to employment. The novelty of this study lies in utilizing the internationally standardized InSCI survey (an ICF-based tool) to quantify these self-reported barriers specifically within the US context. By integrating sociodemographic, injury-related and policy-specific variables into a single multivariate model, this study uniquely links subjective barriers directly to US-specific systemic factors. This study aims to examine sociodemographic and injury characteristics, employment outcomes and perceived barriers among individuals with SCI, based on recent data collected from U.S. participants of InSCI.

## 2 Materials and methods

### 2.1 Study design

This study is an observational cross-sectional study within a community-based setting, part of the second (most recent) International Spinal Cord Injury survey (InSCI), focused on the United States sample of SCI participants (16). Four U.S.-based institutions participated in InSCI (one northeast region, one mid-Atlantic, and two southwest regions). Ethics approval was obtained by each participating institution and informed consent was obtained.

### 2.2 Sample and eligibility criteria

The study included individuals with SCI, aged 18 to 65 years, residing in the United States. The age range was defined *a priori* according to the InSCI protocol, consistent with previous InSCI publications (6, 17), and reflects the typical working-age population. Inclusion criteria required that participants be within the specified age range, living outside institutional care, not fully recovered neurologically, and able to provide informed consent. Eligible participants were recruited from 2023 to 2024, identified through hospital databases, specialized rehabilitation facilities, patient organizations, government agencies, and previous study databases. Respondents could complete the questionnaire through several modes (online, by mail, telephone interview, or in person based on local logistics and participant preference). Each participating center was responsible for ensuring adherence to national ethical guidelines and data protection regulations. Participants were recruited using convenience sampling.

### 2.3 Variables

The survey included a total of 75 items covering injury characteristics, sociodemographic information, various domains of daily life activities (e.g., self-care, participation, energy, emotional well-being), and work-related status (paid work). The questionnaire utilized a combination of binary (Yes/No), categorical, and Likert-scale response formats. Employment status was determined by a binary response (‘Yes’/‘No’) to the question ‘Are you currently engaged in paid work?’. This definition includes all forms of paid labor, regardless of hours worked (full-time, part-time, or casual), which distinguishes it from studies defining employment strictly as full-time competitive work. For this paper, the sociodemographic and lesion characteristics were used, along with the work-related information. Barriers to employment were assessed using a pre-defined checklist of potential reasons for employment. Marital status was dichotomized into two categories: ‘living with a partner’ (married or in a partnership) vs. ‘living without a partner’ (single, separated, divorced, or widowed). Vocational counseling was determined by a binary response (‘Yes’/‘No’) to the item inquiring whether the participant had received vocational rehabilitation services following their injury. For analysis purposes, the age range was classified in five different classes (18–29, 30–39, 40–49, 50–59, 60–65 years), the total number of years lived with SCI (time since injury) was classified into four different categories (0–4, 5–14, 15–24, >=25 years), and education into three categories (0–9, 10–16, >=17 years).

### 2.4 Analysis

Descriptive statistics (means, standard deviations, frequencies, and percentages) were used to describe participant characteristics, categorical, and nominal data. In total, 42 cases were removed from the initial 209, based on the inclusion criteria (older than 65 years of age), leaving 167 participants for the analysis. In order to examine the factors associated with paid work engagement, a binary logistic regression was conducted. The predicted variable was paid work status (0 = No, 1 = Yes) and the predictors were sociodemographic (e.g., age group, marital status, education), injury characteristics (SCI type, degree, cause, and time since injury), and support-related factors (vocational counseling, disability benefit).

In order to examine the factors influencing the employment status (dependent variable with two categories: 0 = No paid work, 1 = Yes, at the time of the study) among the participants, a hierarchical binary logistic regression analysis was conducted. Three conceptual blocks were used: sociodemographic characteristics (Block 1), SCI characteristics (Block 2), and employment or support-related variables (Block 3). The independent variables (predictors) were sex, age class, living in urban area, marital status, education class, time since injury, SCI-type and degree, cause of injury, employment status before the injury, receiving vocational counseling, and receiving disability benefit status. Missing data were handled using listwise deletion (including the *n* = 13 cases missing primary employment status data). More specifically, participants with missing values on the dependent variable or any of the predictor variables were excluded from the regression analysis, resulting in a final analytical sample of *N* = 146. The selection of independent variables was guided by the theoretical framework of the InSCI protocol and the ICF model to ensure international comparability, rather than statistical stepwise reduction. The model fit was assessed for every block with the −2 log-likelihood, chi-square, Hosmer-Lemeshow goodness of fit, and pseudo-R^2^ values. Odds ratios [Exp(B)] and 95% Confidence Intervals (CIs) are reported for each independent variable.

All analyses were conducted using SPSS Statistics (version 26), with statistical significance set at *p* < 0.05.

## 3 Results

### 3.1 Sociodemographic and injury characteristics

Overall, 167 people with SCI were included in this analysis. The majority of the participants were males (*N* = 95, 56.89%). Most respondents were between 30 and 39 years old (*N* = 46, 27.54%) The median age was 42 years of age. The vast majority had received at least 10 years of education (*N* = 120, 71.84%). Approximately 60% (*N* = 99) were living in urban areas and 43.11% (*N* = 72) were single. The prevalence of paraplegia was 54.49% (*N* = 91), while 73.05% (*N* = 122) of the respondents reported incomplete SCI. Roughly, one out of five participants (21.56%) has been living with SCI for 0–4 years. Finally, 147 (88.02%) reported traumatic causes of the SCI. The sociodemographic and injury characteristics can be found in Table 1.

**Table 1:** Sociodemographic and injury characteristics of the US-based participants with SCI.

Variable	Response	*N* (%)
Gender	Male	95 (56,89)
	Female	72 (43.11)
Age classes (years)	18–29	28 (16.77)
	30–39	46 (27.54)
	40–49	38 (22.75)
	50–59	27 (16.17)
	60–65	28 (16.77)
Years of overall education	0–9	40 (23.95)
	10–16	72 (43.11)
	≥17	48 (28.74)
	Missing	7 (4.19)
Urbanity	Rural	68 (40.72)
	Urban	99 (59.28)
Marital Status	With significant other	72 (43.11)
	Without significant other	94 (56.29)
	Missing	1 (0.60)
Years lived with SCI	0–4	36 (21.56)
	5–14	57 (34.13)
	15–24	43 (25.75)
	≥25	28 (16.77)
	Missing	3 (1.80)
SCI Type	Paraplegia	91 (54.49)
	Tetraplegia	76 (45.51)
SCI Degree	Complete	45 (26.95)
	Incomplete	122 (73.05)
SCI Cause	Traumatic	147 (88.02)
	Non-traumatic	20 (11.98)

### 3.2 Employment status characteristics

In total, 67.7% of the participants reported that they were engaged in paid work at the time of the study. Only 62 (37.13%) mentioned that they had received vocational rehabilitation services after their SCI, and approximately 50% (*N* = 83) were receiving disability benefits (Table 2). For the individuals who were engaged in paid work, 54.67% were satisfied with the amount of their working hours, and the rest would either like to have more (24%), or less (21.33%) working hours. On the question whether they “receive the recognition they deserve for their work,” only 12% responded that they “Disagree” or “Strongly disagree”. With respect to whether they find their salary adequate, 18.67% reported that they “Disagree”. For the unemployed individuals, there were items considering their willingness or ability to work. More specifically, approximately 70% (*N* = 55) expressed their desire to work, and their ability to work ranged from 1 to 11 h/week (18.75%), 12–20 h/week (25%), up to more than 20 h/week (23.75%). Table 3 shows the responses of the employed participants.

**Table 2:** Before SCI and current employment status characteristics.

Variable	Response	*N* (%)
Job before the SCI	No	41 (24.55)
	Yes	113 (67.66)
	Missing	13 (7.78)
Currently paid work	No	41 (24.6)
	Yes	113 (67.7)
	Missing	13 (7.8)
Vocational rehabilitation services after SCI	No	92 (55.09)
	Yes	62 (37.13)
	Missing	13 (7.78)
Current disability pension	No	71 (42.51)
	Yes	83 (49.70)
	Missing	13 (7.78)

**Table 3:** Self-reported perceptions of work conditions.

Variable	Response	*N* (%)
Preferred number of working hours	More	18 (24.00)
	Less	16 (21.33)
	The same	41 (54.67)
Receiving the recognition they deserve	Strongly agree	23 (30.67)
	Agree	43 (57.33)
	Disagree	8 (10.67)
	Strongly disagree	1 (1.33)
Receiving an adequate salary	Strongly agree	18 (24.00)
	Agree	41 (54.67)
	Disagree	14 (18.67)
	Missing	2 (2.67)

Individuals who did not have paid work at the time of the study were asked, “What are the reasons you are not currently working?” The vast majority (82.5%) reported due to insufficient transportation services, 33.8% due to fear of losing disability benefits, 11.3% due to lack of accessibility to potential workplaces, 6.3% due to lack of assistive devices and 10% because they did not want to work. A few (3.8%) stated that they were still engaged in educational or vocational training and 6.3% reported that the reason for not working was their health condition. No responses were recorded for “Do not know how or where to seek work” or “Do not have the financial need”, or “Parents or spouse did not let me work”. Only 1.3% reported that it was due to personal or family responsibilities, and 1.3% that they could not find suitable work. In total, 51.3% reported only one of the above-mentioned barriers, 37.5% reported at least two, and 3–5 barriers were observed in 8.7% of the responses. The self-reported categorical data of the unemployed participants can be found in Table 4.

**Table 4:** Self-reported barriers to employment.

Variable	Response	*N* (%)
Desire to work	No	22 (27.50)
	Yes	55 (68.75)
	Missing	3 (3.75)
Work ability	Not at all	23 (28.75)
	For 1–11 h/week	15 (18.75)
	For 12–20 h/week	20 (25.00)
	For >20 h/week	19 (23.75)
	Missing	3 (3.75)

### 3.3 Logistic regression analysis

Overall, the regression analysis (Table 5) included 146 out of the 167 participants, who had complete data for the respective variables. Three blocks were used, resulting in three models:

- Model 1: Sociodemographic Characteristics. This model predicted employment status (*χ*^2^ = 40.24, *p* < 0.001, Nagelkerke R^2^ = 0.321). Age classes, marital status, and education level were significant predictors. Participants aged 30–49 had significantly higher odds of being employed compared to those aged 60–65 (ORs = 19.5 and 18.8, *p* < 0.01). Living alone was associated with lower odds of employment (OR=0.23, *p* = 0.018), as was having fewer years of education (10–16 years: OR=0.21, *p* = 0.013).
- Model 2: Injury characteristics. Adding SCI-related variables further improved model fit (*χ*^2^ change = 16.94, *p* = 0.010; Nagelkerke R^2^ = 0.432). Time since injury emerged as a significant predictor. Respondents within 0–4 years post-injury had substantially lower odds of employment (OR=0.044, *p* = 0.004) compared to those injured ≥25 years ago.
- Model 3: Support and employment variables. This model showed the best fit (*χ*^2^ = 88.50, *p* < 0.001, Nagelkerke R^2^ = 0.606), correctly classifying 81.5% of the cases. The strongest predictor was disability pension status: those not receiving a pension were over 16 times more likely to be employed (OR=16.72, *p* < 0.001). Other variables, including prior employment, vocational counselling, and SCI type, were not statistically significant in the final model.

**Table 5:** Binary logistic regression results for employment status among individuals with SCI (final block model).

Variable	B	SE	*p*-value	OR [Exp(B)]	95% CI (Lower)	95% CI (Upper)
Constant	−1.077	1.111	0.332	0.341	-	-
Sex: Male (ref) vs. Female	0.018	0.531	0.973	1.018	0.360	2.879
Age: 60–65 years (ref)	-	-	-	-	-	-
Age: 18–29	1.417	1.037	0.172	4.127	0.541	31.507
Age: 30–39	2.970	0.857	0.001	19.485	3.635	104.453
Age: 40–49	2.935	0.870	0.001	18.827	3.419	103.681
Age: 50–59	−0.545	0.951	0.567	0.580	0.090	3.740
Rural (ref) vs. Urban	0.497	0.518	0.337	1.645	0.596	4.536
Marital Status: Without partner (ref)	−1.480	0.627	0.018	0.228	0.067	0.777
Education ≥17 years (ref)	-	-	-	-	-	-
Education 0–9	−0.461	0.703	0.512	0.631	0.159	2.502
Education 10–16	−1.551	0.626	0.013	0.212	0.062	0.724
Time since injury: ≥25 years (ref)	-	-	-	1	-	-
Time since injury: 0–4	−3.113	1.091	0.004	0.044	0.005	0.377
Time since injury 5–14	−1.454	0.823	0.077	0.234	0.047	1.171
Time since injury: 15–24	−1.445	0.840	0.085	0.236	0.045	1.224
SCI Type (ref = Tetraplegia)	0.930	0.531	0.080	2.534	0.895	7.171
SCI Degree (ref = Incomplete)	−0.469	0.610	0.443	0.626	0.189	2.071
SCI Cause (ref = non-traumatic)	1.452	0.848	0.087	4.273	0.811	22.505
Employment before SCI (ref = Employed)	−0.764	0.665	0.251	0.466	0.126	1.717
Vocational counseling (ref = Yes)	−0.387	0.558	0.488	0.679	0.227	2.026
Disability benefit: (ref = Yes)	2.817	0.604	<0.001	16.719	5.122	54.566

The Hosmer–Lemeshow test indicated good model fit at each stage (*p*-values > 0.64).

## 4 Discussion

In this study, the sociodemographic characteristics, employment status, and perceived work-related barriers among individuals with SCI living in the U.S. were investigated. The results highlight that employment status is a complex concept depending on various sociodemographic and support-related factors. Modifiable predictors of employment after SCI should be considered and validated, so concrete strategies can be developed to facilitate return-to-work.

Overall, 67.7% of participants in our study reported being engaged in paid work at the time of the survey, a notably high percentage that is consistent with recent findings (18). First, the InSCI survey utilizes a highly inclusive definition of employment (any paid work, including part-time), whereas other studies often measured strictly full-time competitive employment. This high percentage is also likely attributable to recruitment bias due to the convenience sampling. Participants were recruited through rehabilitation hospitals and were more connected to services than the general SCI population. Finally, this high percentage may reflect the adoption and promotion of remote work during the post-COVID era, eliminating the obstacles such as inaccessible transportation. Nevertheless, in a high-income setting like the United States, employment after SCI is shaped by systemic barriers and individual circumstances, reinforcing the need for policies and services that support long-term workforce participation.

The age of the participants was a strong predictor. More specifically, persons that were 30–39 and 40–49 years old had higher odds of employment with predicted probabilities of 80%–85% approximately, in comparison to 15%–20% for those aged 18–29 and 60–65. The above-mentioned age groups had greater chances for work reintegration, while the younger may still be in transition, and the older age group is closer to retirement. Accordingly, the years lived with SCI (otherwise: time since injury) was also a significant independent variable. Individuals with 0–4 years since injury had a predicted employment probability of approximately 4% when compared to those who sustained SCI 15 or more years ago. Hence, early rehabilitation and vocational interventions or support services may be critical during the post-injury period. The marital status was classified into two categories: with a significant other (partner) and without a partner. The individuals without a partner were less likely to be employed than those who had a relationship (approximately 23% vs. 77%). The result may reflect the emotional or financial support from a partner or even the facilitation of commuting. However, it is also important to consider the reverse dynamic: single individuals typically face greater financial pressure to secure independent income. The fact that single individuals in our study still demonstrated lower employment rates, underscores how profound the physical and environmental barriers are without a partner's daily support system. Similarly, education was also a significant predictor, with participants with over 17 years of education having 77% chances of being in paid work. This confirms previously established literature suggesting that higher education allows for better reintegration into the workforce (15). In the systematic review, where the majority of retrieved studies (approximately 70%) were US-based, the authors similarly identified age, time since injury, education, and marital status as critical determinants of employment outcomes.

Concerning the third block of the regression (disability benefit and vocational counseling), the disability benefit status proved to be a substantial predictor. Participants not receiving a disability pension had a predicted probability of employment of approximately 77%, while the recipients of benefits had a probability of 15%. Results raise concerns that disability benefits can be a disincentive for work reintegration within the US. These findings differ significantly from patterns observed in other developed nations. For example, evidence from Canada shows that flexible disability insurance programs can increase participation (19), while findings from Finland show that reducing disability pensions is only partially matched by increased employment (20). As comparative research by De Boer et al. (21) demonstrates, a primary reason for this discrepancy is that while legal criteria for disability may look similar globally, their applications and operationalization differ. First, the US employs a strictly medical evaluation of disability, whereas many European nations evaluate disability using a combination of medical, functional, and rehabilitation-focused criteria. Second, in many international systems, “work disability can be a matter of full or partial disability” (21). In European countries and Canada, these graduated disability pensions allow individuals to work part-time without losing their safety net. Conversely, the US system enforces a strict ‘all-or-nothing’ threshold. Exceeding income limits triggers a sudden ‘benefit cliff,’ stripping individuals of essential healthcare and financial coverage.

Policy initiatives for return-to-work outcomes may help reduce barriers to employment of people with SCI. Variables such as sex, urbanity, SCI type/degree, and cause, vocational counseling, and pre-injury period employment status were not found to be significant by the final model. This could mean that their contribution to the model was mediated by the other factors or was less substantial when all the markers were included.

Our study showed that there are significant barriers to employment for people with SCI that need management and policy-level interventions. Among the various limitations reported by the unemployed subset, insufficient transportation was the most prominent, with approximately 83% reporting it as a major barrier to work. In addition, 11.3% of the respondents reported a lack of accessibility to the workplace. These highlight the lack of accessible transportation options, restricting willing and capable persons with physical limitations from working and integrating into the community, similar to previous study results (22–24).

Approximately one out of three participants (33.8%) mentioned disability benefits (which in the US refers primarily to SSDI and SSI and functions as the equivalent of the ‘disability pension’ in international systems) as a primary barrier to employment (disincentive). Rather than reflecting a lack of motivation, this finding highlights the structural “benefit trap” inherent in US disability policy, where earnings above a threshold can lead to a total loss of cash benefits and essential health coverage (14). This interpretation aligns with our regression results, where participants not receiving benefits were over 16 times more likely to be employed. These data contradict the assumption that unemployment is due to unwillingness to work. The motivation to work or the health condition *per se*, did not seem to be predominant issues of unemployment, since only 10% said they do not want to work, and 6.3% mentioned health reasons. Over 45% reported more than one limitation to work, highlighting that the employment barriers are multiple/cumulative and certainly complex. Furthermore, approximately 70% of the respondents who were not engaged in paid work at the time of the study expressed the desire to work. This gap between the desire to work and actual employment suggests that current policies are failing to support the rights-based framework of the UN CRPD (13), which mandates access to employment as a human right.

Hence, a robust multivariate model integrating the perceived barriers can provide an informed practice/policy and define prioritization of an early post-injury and lower education subgroup, addressing interventions of transportation and access options with disability benefit reforms. The implementation of the ICF-based InSCI survey can provide comprehensive information about the nature of barriers that limit access by persons with SCI or any other health condition to health services and programs.

The implementation of self-reported outcomes, as in the respective study, can add important context to the employment status of individuals with SCI. Objective medical evaluation and clinical indicators are important; nevertheless, self-reported measures provide insights into the perceived barriers, particularly regarding barriers and facilitators to social participation and psychological well-being. The value of this approach extends beyond SCI. For instance, a recent study by Theotokatos et al. (25), using the WHO Disability Assessment Schedule (WHODAS) 2.0, also an ICF-based tool, demonstrated that self-reported disability levels are strongly associated with employment status across welfare applicants in Greece. Although their study considered population with various health conditions, it highlights the importance of integrating self-reported outcomes when evaluating functioning and social participation within a biopsychosocial framework.

Self-reported data for assessing functioning and participation have previously been examined in studies that linked the Patient-Reported Outcomes Measurement Information System (PROMIS) to participation domains and functioning (26). Implementing a holistic approach of evaluation (medical and biopsychosocial) into rehabilitation and employment services can help to identify hindrances, develop tailored vocational interventions, and inform policy-makers on disability benefit management, ultimately promoting equitable workforce participation and improving quality of life for individuals with SCI.

There are certain limitations to this study. The study design was cross-sectional and sampling was of convenience; hence, the results should be interpreted with caution. Longitudinal data may provide a more comprehensive picture of work integration in this population, given the long-term impact of SCI. Nevertheless, the employment status of chronic health conditions, such as SCI, is a complex concept, and latent covariates may be present. In addition, the sample size of the study is small and only a subset of the SCI population living in the US. Convenience sampling is a limitation that also prevents us from generalizing our findings to the SCI population in the US. Additionally, the number of predictor variables included in the regression model is relatively high given the sample size of the unemployed subgroup (*N* = 41). While these variables were included based on their theoretical relevance to the ICF framework, this low events per variable, may increase the risk of model overfitting. This is reflected in the wide confidence intervals for certain predictors (e.g., disability benefits), suggesting that while the direction of the association is robust, the precise magnitude of the effect should be interpreted with caution.

This study highlights the factors associated with employment status among individuals with SCI in the US. Individual and systemic barriers to work integration were found. Early and sustained vocational rehabilitation is crucial for people who sustain SCI to develop their skills and prepare themselves to be part of the workforce. Improving transportation and workplace accessibility is essential to removing environmental barriers. Disability benefit reforms can ensure support, instead of penalizing return to work. Fear of losing essential benefits and support (financial disincentive) makes persons with SCI reluctant to return to the labor market. Rather, those incentives should promote equity, eliminating some of the hindrances they face and advocating for their right to work, increasing their independence in the community, social participation, quality of life, and functioning. Ensuring that individuals with SCI can access employment is not only a health and policy issue but also a human rights obligation under the CRPD, requiring systemic changes to remove barriers and promote participation in the labor market.

## Data Availability

The raw data supporting the conclusions of this article will be made available by the authors, without undue reservation.
